# Supplementary material for: Is this the solution to wellbeing and burnout management for the critical care workforce? A parallel, interventional, feasibility and realist informed pilot randomized control trial protocol
Source: PLoS One. 2023 Apr 27;18(4):e0285038. doi: 10.1371/journal.pone.0285038 (PMC10138256; doi:10.1371/journal.pone.0285038)
Supplement: S2 Table — (PDF) [file pone.0285038.s002.pdf]

HREC Application Form

Achieving wellbeing and managing burnout in critical care health professionals: a parallel, interventional, feasibility and realist informed pilot Randomized Control Trial

ID:4703 Year:2022 Version:6

Project Details

Project Information

All research conducted by, and/or with, SA Health (including Southern Adelaide Local Health Network - SALHN) staff, patients, visitors, premises or data sets needs to be approved by an SA Health Human Research Ethics Committee. Once Ethics approval has been obtained from an SA Health Ethics Committee, please notify us by completing the "Cross-Institutional Approval Form" in the online system.

Teaching & Learning applications can only be submitted for the evaluation of teaching projects for research purposes.

Coursework applications can only cover student projects that are considered low risk and where research results will be disseminated beyond the University and interested parties. This **does not** cover above low risk, Honours, Masters by Research, or PhD student projects.

The World Health Organization's definition for a clinical trial can be found here.

A1. Project Title

Achieving wellbeing and managing burnout in critical care health professionals: a parallel, interventional, feasibility and realist informed pilot Randomized Control Trial

A2. Type of Project

- (•) Research involving human participants
- ( ) Clinical trial involving human participants
- ( ) Teaching & Learning Program evaluation involving human participants
- ( ) Coursework application (Masters by Coursework student projects only)
- ( ) Research only involving existing and de-identified data sets

A3. Anticipated Start Date

The Committee cannot grant retrospective approval so data collection cannot commence until Ethics approval has been granted.

12/09/2022

A4. Anticipated End Date

The first approval period is limited to five years. However, projects can be extended at the end of the approval period if required (subject to approval of annual reports).

30/04/2023

A5. Do you have obtained Ethics approval from another NHMRC registered Human Research Ethics Committee?

- ○ Yes
- ◉ No

A6. Will your project include the following types of research?

- [x] Psychotherapeutic and/or behavioural therapies
- [ ] Health Service changes
- [x] Preventative care strategies
- [x] Educational interventions related to health
- [ ] Collection, or access to, physical samples from human beings (e.g. blood, tissue, cells etc.)
- [ ] Cellular Therapy
- [ ] Ionising and non-ionising radiation
- [ ] None of the above

A7. Will your research be impacted by the following?

For further information about the Defence Trade Controls Act click here.

For further information about the Foreign Influence Transparency Scheme / Foreign Interference click here.

For further information about the Australian Sanctions regime click here.

- [ ] Defence Trade Controls Act
- [ ] Foreign Influence Transparency Scheme / Foreign Interference
- [ ] Sanctions regimes
- [x] None of the above

A8. This research project is for:

- ○ University Research
- ◉ PhD Research
- ○ Masters by Research
- ○ Masters by Coursework
- ○ Honours Research
- ○ Undergraduate studies

A9. Please provide a brief lay summary of the research project.

This research seeks to conduct a pilot Randomised control trial to determine the feasibility and acceptability of intervention versus the control group aimed at improving wellbeing and decreasing burnout amongst critical care healthcare professionals. The project will test weekly debrief sessions alongside an Individualised Management Plan (IMP) for the intervention group, versus only weekly debrief sessions for the control group. This research aims to determine the feasibility and acceptability to inform a larger multi-site randomised control trial.

A10. Will you target participants for whom there are specific ethical considerations?

In accordance with the National Statement on Ethical Conduct in Human Research 2007 (Updated 2018), specific issues arise in the design, conduct and ethical review of research involving the categories of participants identified in this section. Please see Section 4 of the National Statement for further information.

- [ ] Children
- [ ] Indigenous communities
- [ ] People in dependent and/or unequal relationships
- [ ] People unable to give consent for health or other reasons
- [ ] People highly dependent on medical care
- [ ] People with cognitive impairment, intellectual disability or mental illness
- [ ] Women who are pregnant and the human foetus
- [ ] People who are homeless
- [ ] People who are incarcerated
- [ ] People who may be involved in illegal activities
- [ ] Victims of crime
- [ ] Migrants, refugees and asylum seekers
- [ ] Minors 16 years and above
- [ ] People with a cultural and/or religious background
- [ ] People for whom English is a second language
- [x] None of the above

A11. Will the research involve or have an impact on Indigenous communities, including Aboriginal and Torres Strait Islander people?

Research projects involving or impacting Indigenous communities must outline in detail how relevant issues of research design, ethics, culture and language are addressed. Researchers must address the AIATSIS Code of Ethics, and the Ethical conduct in research with Aboriginal and Torres Strait Islander Peoples and communities: Guidelines for researchers and stakeholders must also be addressed. Researchers are also encouraged to read Keeping research on track II, a companion document to Ethical conduct in research with Aboriginal and Torres Strait Islander Peoples and communities: Guidelines for researchers and stakeholders.

- ( ) Yes
- (•) No

Chief Investigator

Chief Investigator Details

The Chief Investigator (CI) has the overall responsibility for the design, conduct, ethical aspects and reporting of a study. The CI is also the key administrative contact for the project and must ensure that all co-investigators and other people involved in the project are fully informed of and comply with relevant policies, guidelines and procedures associated with the project, including intellectual property, confidentiality provisions and granting body’s conditions as required.

Please note: Honours, Masters and Undergraduate students cannot be Chief Investigators. If this project is related to Honours, Masters and Undergraduate research, the CI must be the principal supervisor or course convenor and students must be listed in the Co-Investigator's section.

B1. Chief Investigator's details (PhD student)

Please provide the details of the Chief Investigator below.

Title	Ms
First Name	Nurul

Surname	Adnan
FAN or Student ID	adna0010
College or Portfolio	College of Nursing and Health Sciences
Telephone	0432538983
Email	adna0010@flinders.edu.au

B1.1. Supervisory Panel

Please provide the details of your Supervisory Panel below.

Title	Professor
First Name	Diane
Surname	Chamberlain
FAN	cham0012
College or Portfolio	College of Nursing and Health Sciences
Supervisor Type	Principal Supervisor
Telephone	82013772
Email	diane.chamberlain@flinders.edu.au
Title	Dr
First Name	Claire
Surname	Baldwin
FAN	pren0031
College or Portfolio	College of Nursing and Health Sciences
Supervisor Type	Associate Supervisor
Telephone	+61 8 72218212
Email	claire.baldwin@flinders.edu.au
Title	Dr
First Name	Hila
Surname	Dafny

FAN

College or Portfolio: College of Nursing and Health Sciences

Supervisor Type: Associate Supervisor

Telephone: +61 8 82015121

Email: hila.dafny@flinders.edu.au

B2. Please provide the Chief Investigator's qualifications.

Bachelors of Nursing (Hons)

B3. Please provide detailed information about the Chief Investigator's research experience, including any specific skills or expertise relevant to this project.

- Employed as a Clinical Trials Coordinator in the department of Cardiothoracic Surgery. Conducted phase 4 clinical trial.
- Employed as a Research Nurse Consultant in the department of Rehabilitation, Aged Care, and Palliative Care.
Research mainly focused on inpatient rehabilitation and aged care.

Co-Investigator

Co-Investigator Details

Co-Investigators make a significant contribution to the planning, design, conduct, ethical aspects and reporting of a study. While the Chief Investigator has the overall responsibility for the project, co-investigators must ensure that the project is undertaken in accordance with relevant policies, guidelines and procedures associated with the project, including intellectual property, confidentiality provisions and granting body's conditions as required.

B5. Are there any Co-Investigators?

- (selected) Yes
- No

B5.1. Co-Investigators' details
Please provide the details of your Co-Investigators below.

Title: Associate Professor

First Name: Gavin

Surname: Beccaria

FAN

Telephone: +61 7 4631 2382

Email: Gavin.Beccaria@usq.edu.au

College or Portfolio: External Organisation

Please provide the details of the external organisation.

University of Southern Queensland, School of Psychology and Wellbeing

Is this Co-Investigator a Flinders University student?

- ( ) Yes
- (•) No

Other People

Other People involved in the Project

Other people involved could include mentors, research assistants, statisticians etc. who are not deemed to be co-investigators.

B6. Are there any other persons involved in the project?

- ( ) Yes
- (•) No

Locations

C1. Please provide all locations where the research will be conducted.

All debrief sessions and consults with a Masters in clinical psychologist student (overlooked by an experienced clinical psychologist) will be conducted via the online platform Zoom Video Communications, Inc. 2022. The prescribed individual management plan (IMP) will include Cognitive Behaviour Therapy, administered online, and online reading materials.

C2. Will the project involve access to Aboriginal and/or Torres Strait Islander lands?

- ( ) Yes
- (•) No

C3. Will any research be undertaken overseas?

- (•) Yes
- ( ) No

C3.1. Will this research project involve an International Survey only?

○ Yes
◉ No

C3.2. Will any of the researchers listed in this application be travelling to overseas countries?

○ Yes
◉ No

C3.2.1. As researchers will not be travelling overseas, please outline in detail where and how the research will be undertaken.

Researchers will recruit international participants from New Zealand via participant information flyers distributed by The Australian and New Zealand Intensive Care Society (ANZICS) and The Australian College of Critical Care Nurses (ACCCN). Potential participants interested in participating are required to register their interest via the 'registration and eligibility check form', located on the study's flyer. The intervention group will be administered virtual debrief an individual management plan (once off one-to-one consult) and debriefing which will be conducted virtually/online. The control group will be administered virtual debriefing. All scale measures, questionnaires, and journaling will be administered through the online platform, Qualtrics.

EU Data Collection

If data will be collected within the European Union, it must be collected and stored in accordance with the EU General Data Protection Regulation (GDPR). For further information please see here: https://ec.europa.eu/info/law/law-topic/data-protection_en

Funding

D1. How will your research project be funded?

- [ ] Internal Funding
- [ ] External Funding
- [x] Other Funding (e.g. in-kind support, private funding etc.)

If "Other Funding", please provide more information.

Student research maintenance ($4,000), In-kind support.

D2. Are there any special conditions placed on funding (for example IP rights, data access and storage)?

○ Yes
◉ No

Conflicts of Interest

Conflicts of Interest

Conflicts of interest must be disclosed at the start of a project. Types of activities that can lead to conflicts of interest include, but are not limited to, consultancies, membership of committees, participation in boards or advisory groups and affiliation with or financial involvement in any entity with a direct interest in the subject matter of the research.

Please note that all conflicts of interest must also be disclosed in the Participant Information Sheet.

D3. Will the funding, administrative and/or commercial IP arrangements place any person involved in this project in a conflict of interest?

○ Yes

◉ No

D4. Do any of the researchers have any pre-existing relationships with potential participants?

○ Yes

◉ No

D5. Will there be any constraints on publication?

○ Yes

◉ No

Aims and Justification

E1. What are the aims of the research project?

The aim of this research project is to evaluate two different evidence-based models of individual-focused interventions that seek to impact burnout levels and promote engagement and wellbeing in critical care healthcare professionals.

Primary objectives are as follows:
1. Determine if participants can be recruited, enrolled, and retained
2. Determine if participants can adhere to all components of the intervention

Secondary objectives are as follows:
1. Examine the extent to which participants who receive the intervention have increased levels of wellbeing (and other positive measures) compared to those who do not receive the intervention
2. Examine the extent to which participants have decreased burnout, negative affect (other negative measures) compared to those who do not receive the intervention
3. Examine the extent to which participants who receive the intervention have improved resilience, emotional intelligence, anxiety, and depression compared to those who do not receive the intervention.

E2. Please provide a justification for your research based on a literature review.

Literature Review:
High burnout amongst healthcare professionals have dramatically escalated within the past year due to the rapid dispersion of COVID-19 (Jalili, Niroomand, Hadavand, Zeinali, & Fotouhi, 2021). Nurses and doctors are exposed to unusually high work hours, prolonged working days, and increased levels of work overload and exigency (Silva-Gomes & Silva-Gomes, 2021). Burnout affects healthcare professionals on a national scale even before the COVID-19 pandemic, with statistical reporting of 25% to 75% prevalence rates amongst healthcare professionals worldwide (Dobson et al., 2021; Elghazally, Alkarn, Elkhayat, Ibrahim, & Elkhayat, 2021). Highly stressful professions such as healthcare professionals within critical care environments are more susceptible to developing burnout due to the stressful nature of the workforce (Moss, Good, Gozal, Kleinpell, & Sessler, 2016). Continuous exposure to stress can lead to increased rates of absenteeism, distress, compassion fatigue, and burnout, which eventually compromises the quality and safety of patient care provision (Bateman et al., 2020; Saravanabavan, Sivakumar, & Hisham, 2019). Individual-focused interventions aimed at implementing change such as through mindfulness promotion and wellness curriculum are not consistently sufficient to confront severe burnout symptoms because of contextual factors and mechanisms (Bateman et al., 2020; Gunasingam, Burns, Edwards, Dinh, & Walton, 2015). These factors, for example, gender, age, experience, and marital status differ across individuals, which consequently produces different effects of the intervention. Therefore, the ability to develop an individual-focused intervention that considers such factors can be promising as it provides a more realistic and reliable reporting of outcomes, which can be easily implemented within the 'real world' setting.

Justification:
An umbrella review was conducted to investigate the existence and effectiveness of individual-focused interventions for critical care healthcare professionals. The review reported insufficient evidence for the specific population, instead, reporting on individual interventions for general healthcare professionals. Thus, an expert opinion using realist evaluation was conducted (based on the umbrella review from which a program theory was developed) to determine what individual interventions might be effective, why, in what context, and to what extent, where results were used to refine the program theory. This research project aims to use the program theory as a framework in conducting a pilot randomised control trial (RCT). The pilot RCT seeks to determine the feasibility and acceptability of two evidence-based models of individual-focused interventions to improve wellbeing and decrease burnout amongst critical care healthcare professionals. The ability to provide a solution for this chronic endemic can potentially address and minimise negative personal and work-related consequences of burnout (i.e. poor quality of life, decreased job satisfaction) (De Hert, 2020).

Reference:

Bateman, M. E., Hammer, R., Byrne, A., Ravindran, N., Chiurco, J., Lasky, S., . . . Zu, Y. (2020). Death Cafés for prevention of burnout in intensive care unit employees: study protocol for a randomized controlled trial (STOPTHEBURN). Trials, 21(1), 1-9.
De Hert, S. (2020). Burnout in healthcare workers: prevalence, impact and preventative strategies. Local and regional anesthesia, 13, 171.
Dobson, H., Malpas, C. B., Burrell, A. J., Gurvich, C., Chen, L., Kulkarni, J., & Winton-Brown, T. (2021). Burnout and psychological distress amongst Australian healthcare workers during the COVID-19 pandemic. Australasian Psychiatry, 29(1), 26-30.
Elghazally, S. A., Alkarn, A. F., Elkhayat, H., Ibrahim, A. K., & Elkhayat, M. R. (2021). Burnout Impact of COVID-19 Pandemic on Health-Care Professionals at Assiut University Hospitals, 2020. International Journal of Environmental Research and Public Health, 18(10), 5368.
Gunasingam, N., Burns, K., Edwards, J., Dinh, M., & Walton, M. (2015). Reducing stress and burnout in junior doctors: the impact of debriefing sessions. Postgraduate Medical Journal, 91(1074), 182-187.
Jalili, M., Niroomand, M., Hadavand, F., Zeinali, K., & Fotouhi, A. (2021). Burnout among healthcare professionals during COVID-19 pandemic: a cross-sectional study. International Archives of Occupational and Environmental Health, 1-8.
Moss, M., Good, V. S., Gozal, D., Kleinpell, R., & Sessler, C. N. (2016). A critical care societies collaborative statement: burnout syndrome in critical care health-care professionals. A call for action. American journal of respiratory and critical care medicine, 194(1), 106-113.
Saravanabavan, L., Sivakumar, M., & Hisham, M. (2019). Stress and burnout among intensive care unit healthcare professionals in an Indian tertiary care hospital. Indian journal of critical care medicine: peer-reviewed, official publication of Indian Society of Critical Care Medicine, 23(10), 462.
Silva-Gomes, R. N., & Silva-Gomes, V. T. (2021). COVID-19 pandemic: Burnout syndrome in healthcare professionals working in field hospitals in Brazil. Enfermería Clínica (English Edition), 31(2), 128.

Methodology

E3. Please describe the research approach and methods in more detail.

Participants:

Participants will be recruited through the Australian and New Zealand Critical Care Society (ANZICS) and the Australian College of Critical Care Nurses (ACCCN). The eligibility criteria are as follows: (1) Participants are more than 18 years of age, (2) currently practising within an Australian or New Zealand critical care setting as a registered health professional, Physicians, Nurses, Allied Health, and (3) have access to a personal computer or device with camera and microphone. Potential participants will be excluded if they (1) are under any type of work compensation claims, and (2) have contradictions to any of the interventions in both control and intervention groups. The trial will exclude participants that are considered as 'high risk' of self-harm or psychological harm, determined at the registration phase, assessed using the Impact of Event Scale-Revised (located in the registration form).

Recruitment:
Participants interested in participating are required to complete the 'registration and eligibility check' form located on the study's flyer and website (Qualtrics XM, 2022). An experienced clinical psychologist will screen the participant's 'eligibility check' form (located in the registration form) to determine if participants are safe to participate in the study. Participants that are considered 'high risk' will be on-referred and provided with appropriate acute management. Those confirmed to meet the eligibility criteria and have submitted a completed consent form will then be randomised into a control or intervention group. After randomisation, participants will be sent a set of online surveys that measures their baseline, which will need to be completed – this includes 'Demographic Information', 'Scale Surveys', and 'Journal' entry'.

The intervention group will also be asked to undertake a one-to-one consult with a Master in clinical psychologist student (overlooked by an experienced clinical psychologist) about the participant's (1) lifestyle behaviour assessment (sleep, healthy eating, and exercise) and (2) mental health assessment. The consult will be conducted once-off via the online platform Zoom Video Communications, Inc. 2022. Based on the consultation and baseline psychometric scale survey results, the panel of experts will use their normal scope of practice and devise an individual management plan that suit the individual's needs.

Intervention:
Two intervention arms -

a) Intervention Group:
The Individualised Management Plan (IMP) and debriefing sessions will be administered to the intervention group on a weekly basis for 6 weeks. Participants will be allocated one or a combination of IMP, examples include cognitive behaviour therapy and resource handouts (that focus on sleep, diet, and physical activity). Details for debriefing will be explained below - intervention and control group debriefing will be separate.

b) Control Group
The control group will include weekly (for 6 weeks) debrief sessions administered virtually. A Master in clinical psychologist student (overlooked by an experienced clinical psychologist) will run the 60 minutes debrief session. The debrief session will be held as a group-based session with approximately 10 participants in each group. If participants miss their debrief session, a make-up session will be available – by joining other debrief groups. During each session, the clinical psychologist will ask a range of open-ended questions to participants and encourage engagement between participants.

Website:
The trial website can be accessed by potential participants, where it will be used as the main route of recruitment, completing surveys, booking into debrief sessions, and instructions for IMP.

Outcome Measures:
Scale surveys (qualtrics) will be meaured at baseline, post-intervention, 1-month, and 3-month follow-up. Journaling (qualtrics) will also be conducted weekly throughout the intervention rollout phase. A feedback survey will also be administered post-intervention to obtain further data about the intervention's feasibility and acceptability.

(1)Primary Outcomes - feasibility of recruitment, measure, adherence to the intervention, and acceptability of the intervention.

(2) Secondary Outcomes - scale surveys (measured at baseline, post-intervention, 1-month, and 3-months). Scales used (approximately 1 hour):
1. The Utrecht Work Engagement Scale
2. Attrition Turnover Inventory
3. Maslach Burnout Inventory
4. Practice Environment Scale
5. Problem Solving Inventory
6. Depression, Anxiety, and Stress Scale
7. Impact of event scale
8. Positive and Negative Affect Schedule

Qualitative measures include online weekly journaling, where participants are to provide an open-ended answer to the question: 'I (don't) feel burned out' and 'Why have I (not) adhered to the intervention?' (average completion time 8 minutes).

Sample size:

As this is a feasibility study, there will not be a formal sample size calculation, however, we expect a minimum of 55 participants in each group (intervention and control)

Blinding and Randomisation:
Participants will be randomly allocated into the control and intervention groups in a 1:1 ratio at the pre-baseline phase. Block randomisation will be done by a computer-generated random number list prepared by an investigator with no clinical involvement in the trial. After the participant’s consent is obtained, the investigator (with no clinical involvement) will be contacted – independent of the recruitment process for allocation consignment. Although participants allocated to the intervention group were aware of the allocated arm, outcome assessors and data analysts were kept blinded to the allocation.

Adverse Events:
A clinical psychologist will assess baseline results for the safety of participants. If the trial clinical psychologist identifies, or if the participant voices any psychological, emotional, or physical risks to themselves at any point during the study, the clinical psychologist will provide make a referral to the participant’s workplace or personal psychologist or Beyond Blue Ltd or a psychiatrist or emergency mental health services within 12 hours and followed up daily until the participant is receiving appropriate care. The participant will then be automatically withdrawn from the trial and will be reported as an adverse event. At the initial registration, if the clinical psychologist identifies that a potential participant is ‘high risk’, researchers will exclude the participant and follow the required adverse event processes.

Addition:
We would also like to conduct one-on-one semi-structured interviews with participants at the end of the intervention and ask questions surrounding feasibility and user-perceived acceptability. Examples of questions will include what areas can be improved, what went well, and what changes they would like to see done in future studies. The semi-structured interviews will run for a maximum of 30 minutes per session, which will be conducted via zoom platform. Participants that complete the one-on-one semi-structured interviews will be given a $30 honorarium with respect to their additional time and effort in attending the semi-structured interview.

E4. Do you intend to withhold or disguise the purpose of the research in any way?

- ○ Yes
- ◉ No

Research Instruments

E5. Which of the following instruments will be used in your research project?

- ☐ Hard copy questionnaire
- ☑ Electronic questionnaire
- ☑ Semi-Structured Interviews
- ☐ Workshop
- ☐ Overt Observations
- ☐ Video recordings
- ☐ Movement tracking
- ☐ Performance tests
- ☐ Already existing and de-identified data set
- ☐ Telephone/verbal survey
- ☐ Focus Groups
- ☐ Structured Interviews
- ☐ Covert Observations
- ☐ Photographs
- ☐ Audio recordings
- ☐ Creative, artistic or design process
- ☐ Ethnography
- ☑ Other

Please upload the questionnaire/s.

Documents

Type	Document Name	File Name	Version Date	Version	Size
Default	Weekly Journaling	Weekly Journaling.docx	28/02/2022	1	12.9 KB
Default	Intervention Feedback	Intervention Feedback.docx	31/03/2022	1	13.4 KB
Default	Registration_Form	Registration_Form.docx	21/11/2022	2	28.0 KB
Default	Questionnaire (Psychometric Scales)	Questionnaire (Psychometric Scales).docx	21/11/2022	2	48.8 KB

Please upload the interview questions.

Documents

Type	Document Name	File Name	Version Date	Version	Size
Default	Semi-structured Feedback Questions	Semi-structured Feedback Questions.docx	21/11/2022	1	13.1 KB

Will interview participants be given the opportunity to review and edit interview transcripts?

If Yes, please ensure that participants are given information about this in the Information Sheet and Consent Form.

- (x) Yes
- ( ) No

If "Other", please provide more information.

Field-notes (note taking) during all debrief sessions.

Potential Benefits

E6. What are the benefits of the research project?

Continuous exposure to burnout amongst critical care healthcare professionals can lead to increased rates of absenteeism, distress, compassion fatigue, and burnout, which eventually compromises the quality and safety of patient care provision. Suicide rates have also increased in this group during COVID-19. The ability to develop an intervention that can cater to contextual factors and mechanisms of the individual can provide a solution for this chronic endemic and minimise the negative personal and work-related consequences of burnout.

E7. What research product will be created by this research project?

- [ ] Book(s)
- [ ] Book Chapters(s)
- [ ] Commercial Product(s)
- [x] Conference Paper(s)
- [x] Journal Article(s)
- [ ] Non-traditional research outputs (eg. exhibitions, performances etc.)
- [ ] Report(s)
- [ ] Therapeutic Product(s)
- [x] Thesis
- [x] Other

If "Other", please provide more information.

Proposal for a larger Randomized Control Trial and Grant application.

Recruitment Methods and Participant Groups

F1. Will you, or a third party, recruit participants for this project?

(•) Yes
( ) No

F1.1. Will you, or a third party, recruit any Flinders University undergraduate students?

( ) Yes
(•) No

F1.2. Participant Categories and Recruitment Methods
Please provide information about your participants and recruitment methods below.

Participant Category
For multiple participant categories, please use the "Add Another" button.

Australian registered critical care healthcare professionals (physicians, nurses, and allied health)

Recruitment Method

Snowballing Technique

Estimated Sample Size

110

F1.3. Please expand on the recruitment process and outline in more detail how participants will be recruited.

Participants will be recruited via participant information flyers distributed by critical care societies from Australia and New Zealand (organisation membership, distribution list – allowing for snowballing effect). The sample of critical care healthcare professionals invited to participate will include those who are subscribed to the critical care societies. Participants (from Australia and New Zealand) will be recruited via participant flyers and invitations distributed by critical care societies and colleges using organisational membership and distribution email lists, and social media pages, allowing for snowballing effect. Individuals who are interested in participating are required to complete the registration form and eligibility form via the study flyer.

F1.4. Please upload all recruitment materials, including flyers, introductory emails, verbal scripts, etc.

Documents

Type	Document Name	File Name	Version Date	Version	Size
Default	Flyer Expression of Interest	Flyer Expression of Interest .docx	25/04/2022	1	224.7 KB

F1.5. Will you need to obtain external permission to access participants?

- ○ Yes
- ◉ No

F1.6. Will any of the recruitment information/documents need to be translated into another language?

- ○ Yes
- ◉ No

Participant Consent and Withdrawal

F2. How will participants be able to provide informed consent?

- ☐ Verbal Consent
- ☑ Written Consent
- ☐ Online consent
- ☐ Opt-Out consent
- ☐ Waiver of consent
- ☐ No consent required (existing dataset)

F2.1. Please upload a copy of the Participant Information Sheet and the Consent Form.

Documents

Type	Document Name	File Name	Version Date	Version	Size
Default	Information Sheet and Consent Form	Information Sheet and Consent Form.docx	21/11/2022	2	247.2 KB

F2.2. Please outline in more detail how participants will be able to provide informed consent and how you will ensure that participation is voluntary.

Participants will be notified in the Participant Information Sheet that they are able to withdraw from the trial at any point in time throughout the trial. They will not be expected to complete further scale surveys once withdrawn. Participants will also be notified of the process of withdrawal on the Participant Information Sheet - that is to write an email to the listed email address stating their withdrawal. Participants do not need to provide a rationale for withdrawal and there will not be any penalty.

F2.3. Please outline in detail how participants will be able to withdraw from the research project without penalty and without feeling discomfort.

Participants can email the provided trial email address stating to withdraw without any rationale for withdrawal. The Participant Information Sheet will state that there will not be any penalty or neither will questions be asked regarding withdrawal. Instances where participants who decide to withdraw but do not email the researcher will be contacted by the research team (either via email or phone call) to clarify their withdrawal.

Remuneration and Post Participation

F3. Will any payment, recognition of contribution or compensation be provided to participants?

- (x) Yes
- ( ) No

F3.1. Remuneration of Participants

Please provide information about remuneration of participants below.

Please indicate what type of compensation will be provided.

Voucher

Please provide the monetary value (in AUD).

30

Please explain why this monetary value has been chosen.

For completion of a one-on-one semi-structured interview at the end of the intervention to gain feeback on what went well, areas for improvements, and what they would like to see being done in futrure research. Due to the additional of time commitment and effort, we would like to provide a honorarium for participants.

F4. Will you provide any feedback to participants?

- (x) Yes
- ( ) No

F4.1. Please advise how feedback will be provided.

Feedback will be provided by emailing participants a copy of a short summary of the research outcomes and the published research project.

F5. Will feedback be provided to any organisations, schools and/or people who commissioned or have an interest in this project?

- ○ Yes
- ◉ No

F6. Will a transcription service be used?

- ○ Yes
- ◉ No

Potential Burdens and Risks

G1. Please indicate the possible risk categories to the research team.

- ☐ Physical harms
- ☑ Psychological harms
- ☐ Legal harms
- ☐ Other

G1.1. Please explain the risks in more detail and provide strategies for minimising these risks.

Clinical Psychologist Masters students will be conducting the consults and may be at risk of psychological harm. This will be minimised by ensuring that a clinical psychologist with experience overlooks the students. There will also be a number of clinical psychologist masters students involved in the consultation phase, which will decrease exposure to potential risks of psychological harm.

G2. Please indicate the possible risk categories to participants.

- ☐ Physical harms
- ☑ Psychological harms
- ☐ Social harms
- ☐ Economic harms
- ☐ Legal harms
- ☐ Invasion of Privacy
- ☐ Devaluation of personal worth
- ☐ Risks specific to Indigenous communities, including Aboriginal and Torres Strait Islander people
- ☐ Other

G2.1. Please explain the risks in more detail and provide strategies for minimising these risks.

Psychological harm - conducting debriefing and cognitive-based therapy may expose participants to potential risks of psychological harm. This can be minimised by having a clinical psychologist with accreditation and experience in conducting the interventions supervising the clinical psychologist masters students.

G3. Please indicate the possible risk categories to others not participating in the research project.

- [ ] Physical harms
- [ ] Psychological harms
- [ ] Social harms
- [ ] Economic harms
- [ ] Legal harms
- [ ] Invasion of Privacy
- [ ] Devaluation of personal worth
- [ ] Risks specific to Indigenous communities, including Aboriginal and Torres Strait Islander people
- [x] Other

G3.1. Please explain the risks in more detail and provide strategies for minimising these risks.

Not applicable

Data Collection

H1. What type of data will be collected?

If health and medical information will be collected from the Aboriginal and Torres Strait Islander community in South Australia, an Ethics application must also be submitted to the Aboriginal Health Council of South Australia (in addition to this application).

- [ ] Non-personal information
- [x] Personal information
- [x] Sensitive information
- [x] Health and Medical information
- [ ] Information about the health of Aboriginal and Torres Strait Islander people

H1.1. Please advise what kind of personal and/or sensitive information will be collected.

Name, email address, gender, age, education, employment role, employment pattern, place of work, family responsibility affecting working capacity, and factors influencing participants to leave the health industry.

H2. Does the project require the use or disclosure of information from a Commonwealth agency?

- ( ) Yes
- (•) No

H3. Will health or medical information be sought from a private sector organisation or health service funded by the State Department of Health?

- ○ Yes
- ◉ No

Data Storage & Access

H4. Please outline how long the data, including personal information and contact details, will be stored and used.

- ◉ At least five years from the date of publication
- ○ At least seven years from the date of publication if the research involves a South Australian Government Department
- ○ Permanently if the data relates to work that has a community or heritage value, preferably within a national collection
- ○ (For Coursework Applications ONLY) At least 12-months after the completion of the project if the research project is for assessment purposes only, such as class research projects
- ○ Other

H5. In what format will the data be stored?

- ☐ Hard-copy
- ☑ Electronic copy
- ☐ Artefacts
- ☐ Other

H6. Will the data be stored at a location external to Flinders University?

- ○ Yes
- ◉ No

H7. Who will have access to the data?

- ○ Only personnel listed in this application
- ◉ Other researchers than those listed in this application

H7.1. Please provide the details of the other researchers and advise why access is required.

This pilot Randomised Control Trial will inform a larger trial. Access of de-identified data is required as investigators plan to include other researcher's into the larger trial. Access would be beneficial to collaborate and design the larger trial to ensure quality and comprehensiveness.

H8. Do you intend to use the data in future research projects?

Participants must be able to consent to the use of their data in future research projects. This must be clearly stated in the Participant Information Sheet. Please note that the data cannot be used past the data retention period as outlined in H4.

- ◉ Yes
- ○ No

Data Sources & Identifiability

H9. What sources of information will be used in this project?

- [x] Individual participants
- [ ] Relatives or associates of participants
- [ ] Medical/health/mental health records
- [ ] Electoral Roll
- [ ] Law enforcement agency
- [ ] Public Sector organisation
- [ ] Private Sector organisation
- [ ] Publicly available database
- [ ] Privately available database
- [ ] Internet
- [ ] Other

H10. Will the raw data be individually identifiable, re-identifiable or non-identifiable?

- [x] Individually identifiable
- [ ] Re-identifiable
- [ ] Non-identifiable

H10.1. Please outline in detail how individually identifiable and/or re-identifiable data will be decoded and stored to protect the confidentiality and privacy of participants.

Identifiable data (i.e. psychometric scale surveys) will be stored on an R-Drive (Flinders University managed on-site Enterprise Storage) secured by Flinders University.

H11. Will you release identifiable information or will other persons be able to identify participants in your published research outcomes?

( ) Yes, but only with the participants' consent
(•) No

Documents to upload

I1. Do you have any additional attachments to upload?

( ) Yes
(•) No

Signature

Declaration

I, as the Chief Investigator or authorised delegate, certify that:

- All information contained in this application is true and accurate.
- I have had access to and read the National Statement on Ethical Conduct in Human Research 2007 (Updated 2018), and that the research will be conducted in accordance with the National Statement and in accordance with the ethical arrangements of the organisations involved.
- I have consulted any relevant legislation and regulations, and the research will be conducted in accordance with these.
- I have, if applicable, provided all collaborators and other persons involved in this research project with access to this application (online or PDF) and will provide them with all future amendments and reports.
- All collaborators and other persons involved in this project are aware of the requirements and conditions and will conduct the research in accordance with these.
- I will immediately report to Research Ethics & Compliance anything which might warrant review of the ethical approval of the proposal.
- I will inform Research Ethics & Compliance, giving reasons, if the research project is discontinued before the expected date of completion.
- I will adhere to the conditions of approval stipulated by the Committee and will cooperate with the Committee's monitoring requirements, including the provision of annual progress reports and final reports as required.

Please ensure you understand each statement and your responsibilities and then select "Certified" below.

☑ Certified

Signature

Signed: This form was signed by Nurul Adnan (nurul.adnan@flinders.edu.au) on 21/11/2022 1:46 PM
