# Supplementary material for: Is this the solution to wellbeing and burnout management for the critical care workforce? A parallel, interventional, feasibility and realist informed pilot randomized control trial protocol
Source: PLoS One. 2023 Apr 27;18(4):e0285038. doi: 10.1371/journal.pone.0285038 (PMC10138256; doi:10.1371/journal.pone.0285038)
Supplement: Attachment — Submitted filename: The manuscript describes the protocol of a two.docx [file pone.0285038.s003.docx]

The manuscript describes the protocol of a two-armed parallel, interventional, feasibility pilot RCT assessing the efficacy of an intervention aimed to improve wellbeing and burnout management of critical care professionals in Australia and New Zealand.

- The theoretical background, interventions, outcome measures and analysis plan are written with sufficient detail, except for the following:
- It is not clear how the intervention was developed, who developed it and whether critical care professionals were involved in the process.
- Page 19, lines 276-277: “Efforts will be made to attain assessments of participants who drop-out of this study to facilitate the intention-to-treat analysis” Please explain.
- Page 22, lines 333-334: “However, an independent Safety Officer will be present to monitor the participant’s safety” Please explain.
- Page 25, lines 408-409: “…use of two follow-up time points in the design of this study can also establish long-term efficacy of the intervention” This sentence is questionable for a 3-month follow-up.

Minor point (typo)

Page 9, line 35: “Burnout and work engagement are negatively associated and influences mental and physical…” should be “Burnout and work engagement are negatively associated and influence mental and physical…”
