# Supplementary material for: Is this the solution to wellbeing and burnout management for the critical care workforce? A parallel, interventional, feasibility and realist informed pilot randomized control trial protocol
Source: PLoS One. 2023 Apr 27;18(4):e0285038. doi: 10.1371/journal.pone.0285038 (PMC10138256; doi:10.1371/journal.pone.0285038)
Supplement: Attachment — Submitted filename: Response to Reviewers Completed.docx [file pone.0285038.s004.docx]

**Response to Reviewers**

| **Reviewer 1 Comments** | **Author Response** | **Change made** |
| --- | --- | --- |
| The manuscript describes the protocol of a two-armed parallel, interventional, feasibility pilot RCT assessing the efficacy of an intervention aimed to improve wellbeing and burnout management of critical care professionals in Australia and New Zealand. The theoretical background, interventions, outcome measures and analysis plan are well detailed, except for the following: | Thank you for the feedback | Summary comment noted |
| - It is not clear how the intervention was developed, who developed it and whether critical care professionals were involved in the process. | Thank you for your feedback. The intervention was developed using the Realist Methodology. Firstly, the authors conducted an umbrella realist review^[1]^ that mapped and evaluated evidence-based individual-focused interventions to improve well-being and decrease burn-out and its symptoms for critical care healthcare professionals. These interventions were analysed using realist evaluation, which enabled the authors to use the study findings and construct a program theory. Due to the presented knowledge gaps within the study, the authors conducted a realist expert opinion interview with critical care experts^[2]^ on a set of pre-defined individual interventions summarized from the umbrella review. Thus, this step provided the opportunity to refine the existing program theory, which was subsequently used to guide the authors in designing the intervention for this pilot Randomized Control Trial. We have included further clarifications in Lines 100-112. | Lines 100-112 |
| - Page 19, lines 276-277: “Efforts will be made to attain assessments of participants who drop-out of this study to facilitate the intention-to-treat analysis” Please explain. | Thank you for your feedback. The intention to treat analysis is the gold standard for randomized control trial, where the authors hope to utilise and apply in this feasibility study. However, on re-reding this sentence, we think we can better explain the concept and improve the flow of the manuscript. Therefore, we have made improvements to the section that describes the justification of sample size (lines 282-287), and, left reference to intention to treat analysis to the analysis section only, where it better fits (lines 374-375). | Lines 282-287, lines 374-375 |
| - Page 22, lines 333-334: “However, an independent Safety Officer will be present to monitor the participant’s safety” Please explain. | Thank you for your feedback. The independent safety officer will be a researcher who is independent of the study team and is involved in data and safety monitoring activities such as ensuring the safety of participants, identifying, and attending to potential adverse events, and monitoring of the trial’s progress and data quality. We have included further clarification in lines 343-346. | Line 343-346 |
| - Page 25, lines 408-409: “…use of two follow-up time points in the design of this study can also establish long-term efficacy of the intervention” This sentence is questionable for a 3-month follow-up. | Thank you for your feedback. To provide further clarification of this sentence, we have revised the sentence to the following “….use of two follow-up timepoints in the design of this study may also provide understanding of immediate and early longitudinal patterns and outcomes of treatment changes of the intervention.” | Lines 419-421. |
| Minor point (typo)   - Page 9, line 35: “Burnout and work engagement are negatively associated and influences mental and physical…” should be “Burnout and work engagement are negatively associated and influence mental and physical…” | Thank you for your feedback. We have deleted the ‘s’ in the word ‘influence’. | Line 35. |
| **Reviewer 2 Comments** | **Author Response** | **Change made** |
| The present study protocol aims to assess the feasibility and user-perceived acceptability of a combined Individual Management Plan (IMP) and Professional Problem-Solving Peer (PPSP) debrief in comparison to informal peer debrief. I found this protocol really interesting, well-described and well-written. Also, I found the choice of the design very appropriate for the study objective. Please find below two very minor issues which should be solved/clarified: | Thank you for the feedback | Summary comment noted |
| - please delete 'psychometric' when mentioning questionnaire/scales. | Thank you for your feedback. We have replaced the word ‘psychometric’ to the word ‘questionnaire’ to ensure consistency in this manuscript. Please find changes in lines 19, 216, 245, 248, 320, 321, 322, 334, 369. | Lines 19, 216, 245, 248, 320, 321, 322, 334, 369. |
| - what do you mean by 'journaling'? and by 'journal entries'? | Thank you for your feedback. To ensure consistency with this manuscript, we decided to use your feedback and re-name ‘journaling’ to journal entry’ (Lines 218, 321, 334). We have provided clarification on what we mean by ‘journal entry’ in Lines 218-219. | Lines 218, 321, 334. Lines 218-219 |
| **Journal Requirements** |  |  |
| 1. Please ensure that your manuscript meets PLOS ONE's style requirements, including those for file naming. | Thank you for your feedback. We have ensured that the manuscript meets the PLOS ONE’s style requirements, including file naming. | Checked for accuracy |
| 2. We note that the original protocol that you have uploaded as a Supporting Information file contains an institutional logo. As this logo is likely copyrighted, we ask that you please remove it from this file and upload an updated version upon resubmission. | Thank you for your feedback. We have removed institutional logos from the Supporting Information file and uploaded an updated version. | Completed |
| 3. Please review your reference list to ensure that it is complete and correct. If you have cited papers that have been retracted, please include the rationale for doing so in the manuscript text, or remove these references and replace them with relevant current references. Any changes to the reference list should be mentioned in the rebuttal letter that accompanies your revised manuscript. If you need to cite a retracted article, indicate the article’s retracted status in the References list and also include a citation and full reference for the retraction notice. | Thank you for your feedback. To our understanding, we have not cited papers that have been retracted. There were no changes made to the reference list. | Checked for accuracy |

1. Published article titled: What are the solutions for well-being and burn-out for healthcare professionals? An umbrella realist review of learnings of individual-focused interventions for critical care
2. Published article titled: What are the essential components to implement individual-focused interventions for well-being and burnout in critical care healthcare professionals? A realist expert opinion
